# Complications of Therapeutic Plasma Exchange in pediatric patients: An experience at a tertiary care hospital

**DOI:** 10.12669/pjms.39.4.7002

**Published:** 2023

**Authors:** Tooba Fateen, Nighat Sultana, Muhammad Sarwar, Nazish Saqlain

**Affiliations:** 1 Dr. Tooba Fateen, FCPS (Hematology), Associate Professor of Pathology, Allama Iqbal Medical College/JHL, Lahore, Pakistan; 2 Dr. Nighat Sultana, FCPS (Paediatric Medicine) Assistant Professor, Paediatric Intensive Care, University of Child Health Sciences (UCHS), The Children Hospital, Lahore, Pakistan; 3 Dr. Muhammad Sarwar, FCPS (Paediatric Medicine) Assistant Professor, Paediatric Intensive Care, University of Child Health Sciences (UCHS), The Children Hospital, Lahore, Pakistan; 4 Dr. Nazish Saqlain, FCPS (Hematology) Associate Professor of Pathology, University of Child Health Sciences (UCHS), The Children Hospital, Lahore, Pakistan

**Keywords:** Therapeutic plasma exchange, GBS, PICU, Neurological disorders, Hematology

## Abstract

**Objective:**

To find the incidence of various complications of therapeutic plasma exchange (TPE) in ICU admitted children and to determine their association with age, gender, blood group and diagnosis of the patients.

**Methods:**

In this observational study, data of 24 patients who underwent 125 sessions of TPE was collected from the Pediatric Intensive care unit (PICU) and Hematology department of The Children’s Hospital, Lahore from December 2020 to November 2021. Age, gender, blood group, indications and complications observed during and after the TPE procedure were documented on a pre-designed proforma. The data was analyzed by using SPSS version 23. Quantitative variables were presented in the form of mean and standard deviation. Qualitative variables like gender, blood groups, indications and complications of plasmapheresis were presented as frequency and percentage. Chi square test was applied for comparison of variables.

**Results:**

Among the 24 patients, 45.8% were of age group five to ten years with mean age of 7.58 years± 2.04 years and male to female ratio of 0.84:1. Guillain-Barré syndrome (GBS) and Neuromyelitis Optica spectrum disorder (NMO-SD) were the most prevalent among the patients who underwent TPE. Most common complication was hypotension (44.9%), others were febrile reactions (11.6%), unstable vital signs (14.5%) and allergic reactions (24.6%). Blood group, clinical condition and diagnosis of the patient showed significant association with the incidence of TPE related complications.

**Conclusion:**

The majority of problems caused by TPE are considered to be minor. Sudden fall in blood pressure, pruritus, urticarial rash and fever are the common adverse consequences among pediatric patients. Blood group and diagnosis of the patient can determine the development of such complications during plasmapheresis procedure.

## INTRODUCTION

Treatment with therapeutic plasma exchange (TPE) involves removing plasma from the blood and replacing it with an isotonic fluid (usually 5% albumin or frozen plasma) in order to maintain the patient’s proper oncotic pressure.^1^ First described in the early 20th century, it became widely available in the early seventies. Neurological diseases make up a considerable share of the new diagnoses that have been added to the list since then.^2^

Three to five different exchange operations are commonly included in a TPE course, which is performed every 24 to 48 hours. Exchange-dependent kinetics explain why this happens in the first place. Depending on the hematocrit, one to two TPV (total plasma volume) equivalents are replaced over the course of several hours in each exchange. Most immunoglobulin G-class autoantibodies are found in tissues and interstitial fluid (the “third space”), which means that numerous exchanges are frequently necessary because only about 30% of the total quantity in the body is contained in the intravascular space.^3^

When it comes to the technical aspects of TPE like establishing a vascular access and volume distribution, children may be at greater risk than adults due to this therapy’s inherent principles. Hemodynamic instability, coagulation problems, and electrolyte imbalances are more common in critically sick patients, making the procedure less effective.

TPE outcome in different pediatric disorders have been explored in previous studies.^4^,^5^ Our study is focused to evaluate the complications of TPE procedure and its safety in severely ill children admitted in PICU. The study’s secondary aim was to examine several characteristics of TPE patients who experienced any complication and tried to establish association of these complications with age, gender, blood group and diagnosis of the patients.

## METHODS

It was an observational study, conducted in PICU and Hematology department of The University of Child Health Sciences, The Children’s Hospital, Lahore from December 2020 to November 2021. The study was approved by the hospital review committee (Reference No. 2020-172 dated 21/11/2020). A total of 24 children of both genders who had 125 sessions of TPE in total, were enrolled after taking written informed consent from the parents/guardians. Patients whose parents refused to participate in the study or rejected the TPE treatment and/or those who had any contraindication of the procedure were excluded.

Age, gender, blood groups (ABO and Rh-D blood group system) and indications of plasmapheresis (including Autoimmune Encephalitis, Chronic inflammatory demyelinating polyneuropathy (CIDP), Guillain-Barré syndrome (GBS), Guillain-Barré syndrome (GBS) with Autonomic Instability, Neuromyelitis Optica Spectrum Disorders (NMOSD) were noted. Blood grouping was done by tube method, using antisera A, B, & D (Lorne UK) for forward grouping and A cells, B cells, and O cells for reverse grouping.

The procedure was observed by a trained ICU nurse, pediatric resident and a hematologist. The vital signs of all patients were noted before, during and after the procedure and any complication during and after the TPE method were also documented on a pre-designed proforma.

The collected data was entered and analyzed by using SPSS version 23. Quantitative variable like age, was presented as mean and standard deviation. Qualitative variables like gender, blood groups, indications and complications of plasmapheresis were presented in the form of frequency and percentage. Chi square test was applied for the comparison between qualitative variables and p value <0.05 was taken as significant. Effect modifiers like age, gender and indications of plasmapheresis, were controlled through stratification.

## RESULTS

In this study, 24 patients admitted in PICU were included who had 125 sessions of TPE in total. Among the participants, 45.8% (n=11) were male and 54.2% (n=13) were female with male to female ratio of 0.84:1. Majority of the patients (45.8%) were between five to ten years of age with mean age ± 2SD of 7.58 years± 2.04 years.

Various indications shows in Table-I of TPE which are categorized with the incidence of complications. GBS and NMO-SD were the most prevalent diagnoses among the plasmapheresis patients. Children diagnosed with Autoimmune Encephalitis and GBS with Autonomic Instability, although less in number but showed adverse events in multiple sessions.

**Table-I:** Categorization of Indications of the TPE sessions (n: 125) with complications.

	Indication of TPE	Pts	Sessions	With complications		Without complications	

				(N)	%	(N)	%
1	Guillain-Barré syndrome (GBS)	15	71	39	55	32	45
2	Neuromyelitis Optica spectrum disorder (NMO-SD)	4	20	10	50	10	50
3	Autoimmune Encephalitis	2	12	10	83.4	2	16.6
4	GBS with Autonomic Instability	1	2	2	100	0	0
5	Chronic inflammatory demyelinating polyradiculoneuropathy (CIDP)	1	10	3	30	7	70
6	NMO-SD with Lower stem transverse myelitis (LSTM)	1	10	5	50	5	50

	Total	24	125	69 (55.2%)		56 (44.8%)	

Likelihood Ratio: 0.006, P-values 0.019*

Incidence of Adverse events among complicated TPE sessions (n=69 )are shown in Table-II. The most common complication seen during TPE sessions was hypotension (31 of 69 sessions, 44.9%).

**Table-II:** Incidence of Adverse events among Complicated TPE sessions

	Incidence of Complications	N	%
1	Febrile Reactions	8	11.5
2	Hypotension	31	44.9
3	Unstable Vital Signs (RR, HR)	10	14.4
4	Urticaria	17	24.6
5	Infection	1	1.44
6	Difficult IV Cannula Access	2	2.89

	Total	69	100

Stratification of the TPE shows in Table-III associated complications according to age, gender and blood groups. Among these characteristics, age and gender showed no significance with the occurrence of complications during TPE procedure. However, blood group B D-positive and O D-positive showed statistical significance in this regard.

**Table-III:** Stratification of the TPE related Complications according to age, gender and blood groups.

Variable		Complications (n=69)		No complications (n=56)		P value

		N	%	N	%	
Age (years)	0-5	19	27.5	26	46.4	0.08
	5-10	35	50.7	22	39.3	
	>10	15	21.7	8	14.3	
Gender	Male	28	40.6	17	30.4	0.23
	Female	41	59.4	39	69.6	
Blood Group	A+	6	8.7	15	26.8	0.001*
	B+	33	47.8	29	51.8	
	AB+	7	10.2	1	1.8	
	O+	20	29	4	7.14	
	A-	1	1.4	4	7.14	
	B-	2	2.9	3	5.4	

## DISCUSSION

Therapeutic plasma exchange (TPE) removes and replaces plasma to treat a range of disorders. TPE eliminates pathogenic chemicals (antibodies, immune complexes, cytokines), improving health. The method is utilized in practically all sections of medicine for a variety of disorders, some of which might be life-threatening, and consequences that may not otherwise be considered morbid. TPE should be performed only by experienced staff in specialized centers to minimize the risk of complications.^6^

In our study TPE was performed mainly for the neurological conditions among which Guillain-Barré syndrome (GBS) was the most dominant one encompassing more than half of TPE sessions performed. Other disorders included NMO-SD, autoimmune encephalitis, GBS with autonomic instability, CIDP and NMO-SD with Lower stem transverse myelitis (LSTM). It can also be done for other diseases as well like renal diseases and sepsis.

The most common indications reported in the World apheresis registry are neurological disorders.^7^ The use of plasmapheresis to treat neurological and other immune-based illnesses has been commonplace for some time. In spite of their lack of resources and experience, developing nations have been employing it successfully.^8^ Mazahir et al. reported that most of the indications were renal diseases. The difference in indications of TPE is likely due to difference in each centers’ specific subspecialties, center-specific patient selection criteria and classifications.^9^ According to recent research, neurological conditions are the second most common group for which this therapeutic approach is employed. So, our results are in line with the international research. Likewise in the multicenter study by Paglialonga et al., 67.2% and 20.9% of TPE sessions were performed to treat hematological and neurological diseases respectively.^10^ Hematological centers report various proportions, but hematological and neurological disorders are the most frequent indications as shown by Siddiqui et al.^11^

We found hypotension and urticaria to be the common TPE related complications. The hypotension was not life-threatening and did not require vasopressors. As it is evident from the results that gender and age were not of any significance in the occurrence of the complications. Mokrzycki and Kaplan et al., found urticaria to be the most prevalent adverse response to plasma exchange operations while our study showed around 17 cases (24%) who suffered from urticarial rash.^12^ The treatment was labeled as safe, with majority of problems being modest, readily treatable and short-lived. Because the operations were performed at the patient’s bedside, any adverse responses could be handled quickly.

Our findings are consistent with those of Haque et al., where they showed that hypotension was most frequent finding and it was not proved to be life threatening.^13^ Basic-Jukic et al.^14^ identified TPE-related adverse effects in 10.5% patients. After TPE, 2.7% had abnormal sensations, 2.4% had IV site hematomas, while others had bleeding episodes and allergic reactions. Shemin et al.^15^ identified sequelae in 10% of TPE cases, including fever, urticaria and hypocalcemia. Analysis of 154 TPE sessions in children with neurological disorders, reported development of citrate-induced toxicity and transient hypotension.^16^ Blockage of vascular access (3.6), bleeding (2%), disconnection (1.8%), mild hypotension (1.2%), urticaria (1.2%), nausea, and vomiting (0.8%), were reported by another study.^17^ Brunetta et al.^18^ reported TPE complications in children, including hypotension, pain or paresthesia, transfusion reaction, and face edema. These results were concordant with our findings.

Allergic reactions in our study were reported of mild intensity. However, Huestis et al.^19^ conducted an analysis of 42 patients who passed away as a result of TPE and discovered that the most life-threatening consequences were allergic reactions. The indication for the TPE had a significant impact on the incidence of complications following the procedure as well as the nature of child’s disease.^19^ Children who were diagnosed with anti-NMDA receptor encephalitis had a much higher risk of developing complications, as well as a higher risk of developing pruritus and urticaria.^1^ The results of our research also demonstrated that the indication for undergoing TPE has a considerable impact on the likelihood of experiencing problems as a result of the treatment. Findings of Abbas M et al, showed that in adult age group, delay in getting the definitive treatment and variant of Guillain-Barre syndrome had a significant relationship with presence of complications or poor outcome and it was in line with our findings.20

We found Blood group B Rh-D positive and O Rh-D positive to be associated with TPE complications. But since these blood groups are the most prevalent one among our population so further case-control studies required for this purpose to establish reliable and accurate association.

### The limitations of the study:

included patient selection bias, as majority of patients with neurological disorders were selected. The study was time- bound so only immediate complications were included. Long-term follow-up of these patients who underwent TPE procedure can provide a better information regarding clinical outcome. Multicenter research is also recommended to investigate further the patient and procedure related factors, in context of complications and to provide reliable predictors of such adverse reactions

## CONCLUSION

TPE is a therapy for the purification of blood that is mostly risk-free for use in children. The majority of problems caused by TPE are considered to be minor. Sudden fall in blood pressure, pruritus and urticarial rash are the most often seen consequences. Patient’s diagnosis, current clinical state and blood group play an important role in determining development of complications during the procedure.

### New information:

Hypotension is a major complication and must be dealt cautiously and timely to avoid any life threatening conditions but overall TPE is a comparatively safer and life-saving procedure to be performed in PICU especially when performed earlier in the course of disease.

### Authors` Contribution:

**TF:** Designed & Contributed in manuscript writing. She is also responsible for the accuracy of the work.

**NiS:** Collection & assembly of data.

**MS:** Data Collection, Manuscript writing.

**NS:** Review of manuscript and references.
